# The Obscure Effect of *Tribulus terrestris* Saponins Plus Inulin on Liver Morphology, Liver Fatty Acids, Plasma Glucose, and Lipid Profile in SD Rats with and without Induced Type 2 Diabetes Mellitus

**DOI:** 10.3390/ijms22168680

**Published:** 2021-08-12

**Authors:** Kamila Misiakiewicz-Has, Dominika Maciejewska-Markiewicz, Sylwia Rzeszotek, Anna Pilutin, Agnieszka Kolasa, Paweł Szumilas, Ewa Stachowska, Barbara Wiszniewska

**Affiliations:** 1 Department of Histology and Embryology, Pomeranian Medical University in Szczecin, 70-111 Szczecin, Poland; sylwia.rzeszotek@pum.edu.pl (S.R.); anna.pilutin@pum.edu.pl (A.P.); agnieszka.kolasa@pum.edu.pl (A.K.); barbara.wiszniewska@pum.edu.pl (B.W.); 2 Department of Human Nutrition and Metabolomics, Pomeranian Medical University in Szczecin, 70-204 Szczecin, Poland; dmaciejewska.pum@gmail.com (D.M.-M.); ewastachowska.pum@gmail.com (E.S.); 3 Department of Social Medicine and Public Health, Pomeranian Medical University in Szczecin, 48 Żołnierska Str., 71-210 Szczecin, Poland; pawel.szumilas@pum.edu.pl

**Keywords:** fatty acid, NAFLD, NASH, diabetes, saponins, inulin

## Abstract

Diabetes is a predictor of nonalcoholic fatty liver disease (NAFLD). There are data suggesting that *Tribulus terrestris* (TT) saponins act as antidiabetic agents and protect against NAFLD. The effect of saponins may be increased by fermentable fibers such as inulin. The aim of the present study was to investigate the influence of TT saponins and TT saponins plus inulin on the plasma lipid profile and liver fatty acids of rats with induced diabetes mellitus type 2 (T2DM). The study was performed on 36 male Sprague–Dawley rats divided into two main groups: control and diabetic. Animals of the diabetic (DM) group were fed a high-fat diet and injected with streptozotocin (low doses). Animals of the control group (nDM) were on a regular diet and were injected with buffer. After the injections, the animals were split into subgroups: three non-diabetic (nDM): (i) control (c-C); (ii) saponin-treated rats (C-Sap); (iii) rats treated with saponins + inulin (C-Sap + IN), and three diabetic subgroups (DM): (iv) control (c-DM); (v) saponin-treated rats (DM-Sap); (vi) rats treated with saponins + inulin (DM-Sap + IN). Liver fatty acids were extracted and analyzed by gas chromatography, and plasma glucose and lipids were measured. The study showed significant changes in liver morphology, liver fatty acids, plasma lipid profile, and plasma glucose. In summary, supplementation with TT saponins or saponins with inulin for one month decreased the level of steatosis in rats with induced type 2 diabetes. Moreover, there were favorable effects on the plasma lipid profile in the rats. However, additional supplementation with inulin had a negative effect on liver morphology (with a microvesicular type of steatosis) in the non-diabetes group. Moreover, supplementation with inulin had a negative effect on plasma glucose in both diabetic and non-diabetic rats. These data show that a diet enriched with fermentable fibers reveals different effects in different organisms, and not all sources and forms of fiber are beneficial to health.

## 1. Introduction

Glucose and lipid metabolism are mostly regulated by the liver, adipose tissue, and skeletal muscle. A high-fat diet (HFD) is associated with hepatic fat accumulation and insulin resistance (IRes), which, if left untreated, leads to type 2 diabetes mellitus (T2DM). When plasma glucose levels are high, insulin, a hormone produced by pancreatic beta cells, inhibits gluconeogenesis under physiological conditions, but when peripheral tissues are resistant to insulin, gluconeogenesis in the liver increases, which leads to nonalcoholic fatty liver disease (NAFLD) [1]. This is characterized by an accumulation of lipids in the cytoplasm of hepatocytes (steatosis) in the presence of <10 g of daily alcohol consumption [2]. Therefore, T2DM is an independent predictor of NAFLD [3,4]. With further progression, steatosis develops into nonalcoholic steatohepatitis (NASH), and this may lead to hepatic fibrosis/cirrhosis or even liver cancer [5]. There are many clinical features that are characteristic for patients with both T2DM and NAFLD-improper glucose metabolism, abdominal obesity, an elevated level of triglycerides, low levels of high-density lipoprotein cholesterol (HDL), and elevated blood pressure [4].

Fatty liver disease (FLD) has been classified into three subcategories: NAFLD, alcoholic liver disease (ALD), and fatty liver caused by uncommon causes [6]. Both ALD and NAFLD range from simple hepatic steatosis to steatohepatitis and cirrhosis, but there are differences in many characteristics, such as clinical features or patient outcomes [7]. However, many studies from different fields published in the last 1–2 years have been focused on the metabolic component of FLD with the accumulation of fat in hepatocytes as the common factor of this disorder without paying attention to the causes [8,9]. An International Consensus Panel has suggested changing the NAFLD/ALD dichotomy into “metabolically associated FLD” (MALFD) [10]. This is due to the fact that dysmetabolism and at-risk drinking often coexist. As well as this, it is difficult to assess alcoholic intake, and there is also the possibility of endogenous alcohol production in non-drinkers [11].

Experiments on animals that would reflect particular disorders, such as T2DM and/or NAFLD, in humans is a challenge because there are different diets and experimental protocols. Proper diet composition, drugs, the duration of the experiment, strains, and even the sex of the rats are key factors for the whole experiment. It is known that Sprague–Dawley (SD) rats of the male sex fed with a high-fat diet (HFD) are more susceptible to NAFLD development [12,13].

Natural products from plants, such as saponins, are a great source of medical compounds and may become important tools for healthcare. Saponins belong to a group of amphiphilic glycosides, which contain a sugar chain (glycone) linked to a triterpene or steroidal aglycone (sapogenin) moiety [14]. Saponins have the ability to foam upon shaking because they are composed of sapogenin, which is nonpolar, and side-chain, which is water-soluble [15]. They are mostly produced by plants such as *Tribulus terrestris (TT)*, soybean, lucerne, berseem, yucca, etc., but also by some marine animals such as sea cucumbers, starfish, and some rhizobacteria [16]. Saponins reveal many biological activities: immunostimulatory, hypocholesterolemic, antitumor, anti-inflammatory, antibacterial, antiviral, antifungal, and antiparasitic [17].

One promising plant is TT (Zygophyllaceae). It is an annual plant native to the Mediterranean region, which can now be found in other regions of Europe as well as in Asia, America, Africa, and Australia [18]. There are many substances that have been extracted from the plant: saponins, flavonoids, alkaloids, lignanemides, and cinammic acid [18]. Saponins from TT are a class of steroidal saponins such as: spirostanol, furostanol [19], different derivatives of tigogenin, neotigogenin, gitogenin, neogitogenin, hecogenin, neohecogenin, diosgenin, ruscogenin, chlorogenin, and sarsasapogenin [18]. There are data suggesting many beneficial effects of extracts from TT such as: cardioprotective, anti-microbial, diuretic, cytotoxic, anti-hyperlipidemic, wound healing action, and positive effects on individuals with diabetes mellitus [20].

Saponins are hydrolyzed in the digestive system into sapogenins and sugar. After absorption, they are metabolized, but their level in many tissues (with the exception of the kidney and liver) is lower than in the blood [17]. Hydrolization of saponins is possible in the presence of proper intestinal microbiota. However, under certain conditions, such as obesity and diabetes type 2, the intestinal microbiota is changed [21], which may have a negative effect on the hydrolyzation and absorption of saponins. Stimulation of growth or metabolic activity of certain commensal microorganisms (such as lactobacilli, bacteroides, and bifidobacteria) may be achieved by prebiotics such as inulin, which is a natural nondigestible food ingredient [22] fermented by gut microbiota. Moreover, inulin itself is also associated with a decreased risk of obesity and many metabolic disorders such as T2DM [23,24].

The consumption of medicinal plants and the belief that they reveal positive health effects attract many people for supporting the treatment of different diseases. Although many saponins have possible health benefits, many of them may be toxic, so it is important to explore the influence of particular saponins on organisms. The aim of this study was to test whether inulin might increase the potentially positive effect of TT saponins on plasma lipid profile, plasma glucose, liver morphology, and liver fatty acids in SD rats with and without induced T2DM. While the suspected effect of inulin should be positive, it rather had a negative effect on the liver morphology of control rats. These data show that a diet enriched with fermentable fibers reveals different effects in various organisms, and not all sources and forms of fibers are beneficial to health; thus, it should be approached with great caution.

## 2. Materials and Methods

### 2.1. Animal Care and Maintenance

**Figure 1:**
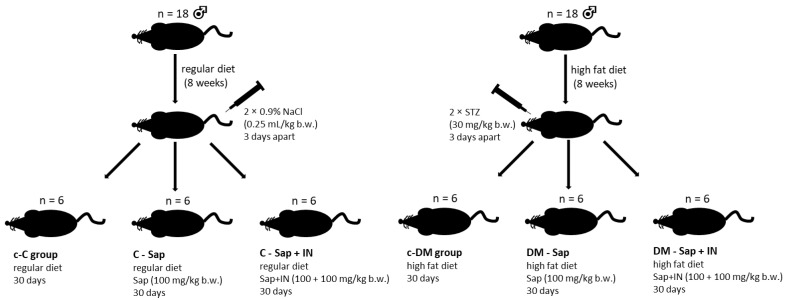
Schema of the experiment.

Thirty-six male Sprague–Dawley (SD) rats were obtained from Animalab (Germany) aged 9–10 weeks. Animals were kept in 12–hour light/darkness cycles in a temperature-controlled environment. All animals were acclimatized for one week on a standard diet before grouping. After that period, the rats were randomly divided into two main groups. Animals of the control group (C; n = 18 rats) were fed with a regular diet, and rats of the high-fat diet group (HFD; n = 18) were fed with a high-fat diet (previously described [25]) to induce insulin resistance. All animals of both groups (C and HFD) were kept in plastic cages (3 rats per cage) and had ad libitum access to water and adequate chow until the end of the experiment. Two months later, the rats were injected twice 3 days apart with: (A) HFD group streptozotocin (30 mg/kg b.w.) (STZ; Sigma-Aldrich) to induce type 2 diabetes (DM group) [25]; (B) control group (c-C group) was injected with 0.9% NaCl in a dose volume of 0.25 mL/kg. Before the second injection, blood glucose levels were tested. Rats with a glucose level greater than 16.67 mmol/L did not receive the second injection. Next, animals of both groups were randomly divided into subgroups (n = 6): 3 subgroups of the non-diabetic group (C) control (C); saponins (100 mg/kg) treated rats (C-Sap); rats treated with saponins (100 mg/kg b.w.) + inulin (100 mg/kg) (C-Sap + IN), and 3 subgroup of STZ-treated rats diabetic group (DM): control (c-DM); saponins (100 mg/kg b.w.)-treated rats (DM-Sap); rats treated with saponins (100 mg/kg b.w.) + inulin (100 mg/kg) (DM-Sap + IN). Substances (saponins and inulin) were administered per os for 30 days, once per day in the form of small pellets made of saponins or saponins + inulin and pressed into a piece of bread. The rats of both control subgroups (c-C and c-DM) received pellets made solely of bread. At the end of the experiment, the animals were terminated under morbital anesthesia (2 mL/kg b.w.) after 12–14 h of fasting. The blood was taken directly from the heart and glycated hemoglobin (HbA1c/HbF), and a lipid blood test was performed. The experiment was approved by the Local Ethical Committee on Animal Testing in Poznań, Poland (approval no. 60/2016). The schema of the experiment is shown in Figure 1.

### 2.2. Histopathologic Evaluation

Part of the liver tissue was fixed in 4% paraformaldehyde embedded in paraffin blocks and sliced into 4 µm sections. The liver sections were then stained with hematoxylin and eosin (H-E) to analyze hepatic steatosis, and Mallory’s trichrome method (Bio–Optica, Milan, Italy) was used to analyze fibrotic changes. Hepatic steatosis and fibrosis were assessed using ten light microscopic fields and scored for the severity of hepatic steatosis and fibrosis according to the criteria of Kleiner et al. (2005) and Xu et al. (2010) [26,27] and as previously described [28]. Grades for hepatic steatosis were: grade 0: steatosis occupying less than 5% of hepatocytes; grade 1: steatosis occupying 6–33%; grade 2: steatosis occupying 34–66%; grade 3: steatosis present in more than 66% of hepatocytes. To examine the staging of hepatic fibrosis, the following criteria were used: 0: without fibrosis; 1: mild, zone 3, perisinusoidal; 2: moderate, zone 3, perisinusoidal; 3: portal/periportal; 4: bridging fibrosis [26,27].

### 2.3. Isolation of Fatty Acids

Fatty acids were extracted according to the Folch method [29]. A total of 60 mg of liver were homogenized and saponified with 3 mL of methanol:chloroform (1:2). Samples were centrifuged, and 1 mL of supernatant was saponified in a 2 mol/l KOH methanolic solution for 20 min at 70 °C and then methylated with 14% boron trifluoride (2 mL) in methanol under the same conditions. Next, n-hexane (2 mL) and saturated NaCl solution (10 mL) were added. One milliliter of the n-hexane phase was collected for analysis.

### 2.4. Fatty Acid Methyl Esters Analysis

Gas chromatography was performed with the use of the Agilent Technologies 7890A GC System (SUPELCOWAX™ 10 Capillary GC Column (15 mm × 0.10 mm, 0.10 μm)). Chromatographic conditions were as follows: the start temperature was 60 ℃ for 0 min, and it was increased at a rate of 40 ℃/min to 160 ℃ (0 min), then increased at a rate of 30 ℃/min to 190 ℃ (0.5 min) and then increased at a rate of 30 ℃/min to 230 ℃ for 2.6 min, where it was kept for 4.9 min. The total analysis lasted about 8 min. The gas flow rate was 0.8 mL/min (hydrogen was the carrier gas). At the end, commercially available standards were used to identify fatty acids by comparing retention times.

### 2.5. Fatty Acid Indices

Ratios of particular FA were used to calculate activities of the hepatic de novo lipogenic (DNL) index (C16:0/C18:2n-6) [30], stearoyl-CoA desaturase 1 (SCD-1; SCD-16 = C16:1n-7/C16:0 and SCD-18 = C18:1n-9/C18:0 [31], ∆6D (18:3n6/18:2n6), FA elongation = (C18:0 + C18:1,n-9)/C16:0, Elovl-6 (C18:0/C16:0) and Elovl-5 (20:3n6/18:3n6) [32].

### 2.6. Statistical Analysis

Statistical analyses were performed with the use of Statistica 13.1 software, and all results are expressed as mean ± standard deviation. The normality of the distributions of variables was verified by the Shapiro–Wilk Test. A comparison between independent groups was performed using the parametric t-test where data were normally distributed and a non-parametric Mann–Whitney U test for non-normally distributed data. To compare the categorical variables, the chi-squared test was used (MedCalc Statistical Software version 16.4.3). *p*-value < 0.05 was considered to be statistically significant, and *p*-value < 0.08 was considered as a trend toward statistical significance.

## 3. Results

### 3.1. Histological Evaluation of the Liver

Hepatic steatosis and fibrosis

**Figure 2:**
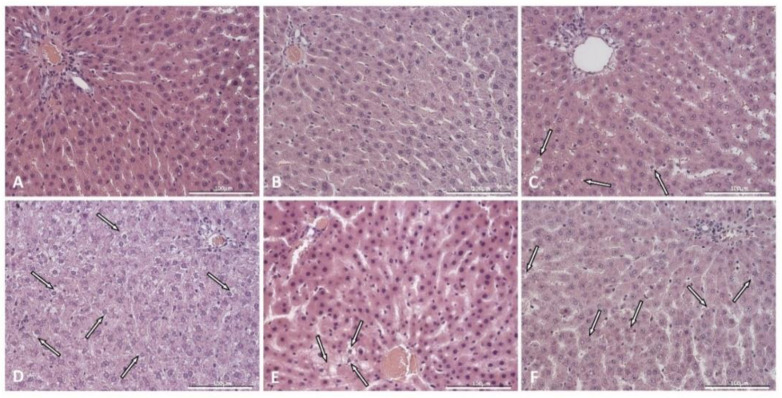
Liver sections from a representative rat from each group: (**A**): control; (**B**): control supplemented with TT saponins; (**C**): control supplemented with TT saponins plus inulin; (**D**): with induced diabetes mellitus; (**E**): with induced diabetes mellitus supplemented with TT saponins; (**F**): with induced diabetes mellitus supplemented with TT saponins plus inulin. Arrows indicate steatosis. H-E × 40. Scale bar: 100 µm.

**Figure 3:**
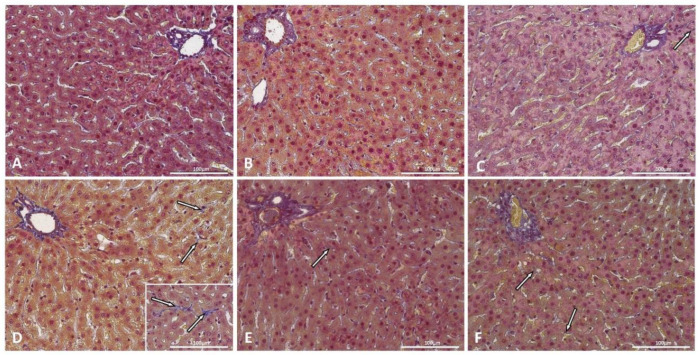
Liver sections from a representative rat from each group: (**A**): control; (**B**): control supplemented with TT saponins; (**C**): control supplemented with TT saponins plus inulin; (**D**): with induced diabetes mellitus; (**E**): with induced diabetes mellitus supplemented with TT saponins; (**F**): with induced diabetes mellitus supplemented with TT saponins plus inulin. Arrows indicate fibrotic changes. Mallory’s trichrome method × 40. Scale bar: 100 µm.

Liver sections of control rats and control rats supplemented with saponins did not show accumulation of lipids in hepatocytes (Figure 2A,B) arranged in plates. Typical hexagonal lobules with a centrally located central vein and with portal spaces at the corners were visible in the liver parenchyma. Interestingly, additional supplementation of inulin was mostly associated with the appearance of small lipid droplets (less than 1 µm) deposited in the cytoplasm of hepatocytes, with a centrally located nucleus-microvesicular steatosis (Figure 2C; Table 1). The livers of rats with induced diabetes were characterized by mixed types of steatosis with microvesicular steatosis as the predominant type (Figure 2D; Table 1). Supplementation with saponins and with saponins plus inulin visibly decreased the number of hepatocytes with lipid deposition (Figure 2E,F; Table 1). Hepatic fibrosis was neither observed in control rats nor in control rats supplemented with saponins (Figure 3A,B; Table 1), but mild perisinusoidal fibrosis was observed in the liver sections of two control rats supplemented with saponins plus inulin (Figure 3C; Table 1). Liver sections of four rats with induced diabetes showed mild perisinusoidal fibrosis, while moderate perisinusoidal fibrosis was present in one rat (Figure 3C; Table 1). Supplementation with either saponins alone or with both saponins and inulin caused that hepatic fibrosis was observed in a decreased number of animals in one animal per each group (Figure 3D,E; Table 1).

### 3.2. The Baseline Plasma Lipid Profile, Glycated Hemoglobin, and Glucose

The baseline plasma lipid profile, glycated hemoglobin, and glucose levels in the control and diabetic groups are shown in Table 2. The comparison of plasma lipid profile has indicated a statistically significant decrease in plasma HDL cholesterol in the control diabetic (c-DM) group compared to the control non-diabetic (c-C) group and an increase (on the border of a statistically significant difference) of the TC/HDL rate in control diabetic (c-DM) compared to control non-diabetic (c-C). There were no significant changes in the level of total cholesterol, LDL, nor triglycerides between c-DM and c-C groups. Supplementation with saponins or saponins + inulin did not cause significant changes in the diabetic nor in the non-diabetic group. One tendency is visible (although not significant): the level of plasma triglycerides is increased in C-Sap + IN compared to the c-C group, while the level of triglycerides is decreased in the DM-Sap + IN group compared to c-DM.

The comparison of glucose levels has indicated a statistically significant increase in the c-DM group compared to the c-C group. In both main groups (control and diabetic), supplementation with saponins did not cause significant changes compared to respective control groups (c-C and c-DM), while supplementation with both saponins and inulin caused a significant increase compared to respective control groups (c-C and c-DM). There were no significant changes in the level of HbA1c/HbF between the c-DM and c-C groups. Supplementation with saponins or saponins + inulin did not cause significant changes in the diabetic nor in the non-diabetic group (Table 2).

### 3.3. Fatty Acid Profile

The study indicated significant changes in the fatty acids of the liver (Table 3). The comparison between the control non-diabetic vs. control diabetic group has revealed statistically significant decreases in the following acids or rates of acids: C16, C17, 18:2n-6 (LA), 18:3n-3 (ALA), total PUFA, PUFA/SFA, total n-6. While total MUFA, DNL index, Elovl-5 index, and Elovl-6 index are significantly increased in the control diabetic group compared to the control non-diabetic group.

In the control group supplemented with saponins, there were statistically significant decreases of C14:1, C18:1n9, C18:1n7, total MUFA, PUFA/SFA, SCD-18 index and a significant increase in C18:0, total SFA and Elovl-6 index compared with the c-C group. A combination of saponins with inulin caused a significant decrease in C12, C17, C18:1n7, and C20:2n6 compared to the c-C group.

Supplementation with saponins caused a significant increase in C12 (on the border of significance), C17 and the rate n-6/n-3 in rats with induced T2DM compared to the control diabetic group. A combination of saponins with inulin seems to strengthen the effect of saponins because it caused significant increases in C12, C14, and C17 and a significant decrease in C18:2n6 (LA), C18:3n3 (ALA), and elongation of the FA index compared to the c-DM group.

## 4. Discussion

Growing consumption of refined foods rich in fats and sugars and a decreased consumption of vegetables and fruits rich in fiber is associated with increased obesity and metabolic diseases such as diabetes type 2, which is often associated with the development of NAFLD [33]. Despite the fact that a balanced diet and physical activity is the best way to avoid many health problems, people use dietary supplements without changing their unhealthy lifestyle. Nowadays, the use of dietary supplements is widespread, and in developed countries, ≤75% of individuals use at least one dietary supplement to alleviate, enhance or influence physiological processes within the body [33]. The goal of such products is to provide beneficial health components naturally present in fruits and vegetables (such as fiber). However, such supplements may have adverse effects.

In the present study, pharmacological induction of type 2 diabetes symptoms through the injection of low doses of STZ and a high-fat diet led to morphological changes in the liver with microvesicular steatosis and fibrosis. Supplementation with T.T saponins did not affect the morphology of the liver of control rats, while in T2DM rats, supplementation with saponins diminished fat content in the liver, reducing the level of hepatic steatosis. This reduction may be associated with the fact that *T.T saponins* show inhibitory activity against *α*-glucosidase [34] and the inhibitory effect of an inhibitor of α-glucosidase on the deposition of fat in hepatocytes in Zucker fatty rats has been previously reported [35].

Steatosis with increased adiposity and insulin resistance can lead to fibrosis, which is linked with an increased number of dying hepatocytes and increased levels of reactive oxygen species. Consequently, the production of adipokines and cytokines is initiated, and this, in turn, activates hepatic stellate cells (HSC) to produce an extracellular matrix [36]. Fibrotic changes with the deposition of collagen fibers in the liver tissue were observed in the present study in rats with induced T2DM. Supplementation with TT saponins caused decreased deposition of collagen in the liver tissue of rats with induced T2DM, while in non-diabetic rats, such supplementation did not cause any changes compared to the control group. The hepatoprotective effect of TT saponins was indicated in the study of Hu [37], which revealed that TT saponins can significantly increase the levels of antioxidants such as superoxide dismutase (SOD) and glutathione peroxidase (GPx), decrease the level of malondialdehyde (MDA) in serum, inhibit expression of Caspase-3 and have a positive effect on the ultrastructure of liver tissue in a mouse model [37].

Surprisingly, in the present study, supplementation with the combination of saponins with inulin caused adverse effects in the diabetic and non-diabetic groups. In the non-diabetic group, supplementation of saponins with inulin caused an accumulation of lipid droplets in hepatocytes resulting in the development of steatosis and the appearance of fibrosis, while in animals with induced T2DM additional supplementation with inulin did not cause significant changes in the level of steatosis.

The present study also showed that induced T2DM caused changes in the plasma lipid profile significantly decreased HDL-C and increased levels of TC/HDL were observed in diabetic rats compared with non-diabetic rats. A similar tendency was observed in the studies of others, where type 2 diabetes was associated with increased triglyceride levels, decreased concentrations of HDL-C, and almost no significant changes in the level of LDL-C [38]. Despite the fact that supplementation with saponins or saponins with inulin did not cause any significant changes in control or in the T2DM group, a certain dependency has been observed. TC and the rate of TC/HDL were not changed in the control group or decreased in the T2DM group after supplementation with TT saponins; however, additional supplementation with inulin caused negative changes in the level of these two parameters (increased TC/HDL and TC) in the control group and did not cause changes (compared to the group supplemented with saponins) in the T2DM group. The effect of inulin on the plasma lipid profile is not clear, and different results are present in the literature [23,39,40]. The situation is even worse when we want to find information about the effects of saponins combined with inulin on the plasma lipid profile because very little data show such results. The study of Yenge et al. [41] indicated that supplementation with the combination of inulin from *Cichorium intybus L*. with saponins from *Sapindaceae* did not exert a positive effect on serum biochemical parameters such as total protein, albumin, globulin, and the ratio of albumin to globulin nor on egg yolk total cholesterol, triglycerides and HDL cholesterol in laying hens [41].

A diet rich in dietary fibers has been associated with many health benefits. Dietary fibers may be resistant to fermentation (insoluble) or may be metabolized by gut microbiota (soluble) such as inulin [42]. Inulin is fermented by intestinal microbiota into short-chain fatty acids (SCFA): acetate, butyrate, and propionate, which play important and beneficial functions such as acting as an energy source for intestinal epithelia and promotion of the differentiation of regulatory T cells, which are anti-inflammatory [43]. It is presumed that a diet supplemented with fermentable fibers improves the health of a person whose diet is rich in highly processed food that do not contain such fibers; however, there are also adverse reports.

Our previous study revealed that additional supplementation with inulin decreased the positive effect of soya isoflavones [28], while the current study showed that the consumption of foods enriched in purified fibers may even have a negative effect in certain conditions on the plasma lipid profile and liver morphology in healthy individuals. Singh et al. [43] documented that prolonged consumption of foods rich in fermentable fiber by mice that have tendencies to dysbiosis had a negative effect on the liver, causing cholestasis, inflammation, and even hepatic cancer [43].

It should also be mentioned that the consumption of inulin is associated with modifications in gut microbiota; however, gut microbiota are different in different species, and the effect of consuming dietary fibers may be different. Despite the fact that the gut microbiota of healthy laboratory rats lack some genera found in humans and have some which are common with mice, the fecal microbiota of rats are closer to humans than mice [44].

In the present study, rats with induced type 2 diabetes mellitus were on a diet enriched in saturated fat. It is well known that a diet rich in saturated fat and carbohydrates causes altered fatty acid (FA) metabolism and elevated triglycerides [45]. Hypertriglyceridemia (elevated levels of TG) is a marker of NAFLD [46], which was also noted in the present study.

Different studies indicate that the development of NAFLD is a result of insulin resistance, while liver fatty acids and the expression level of ELOVL6 (an enzyme that regulates fatty acid composition) are responsible for the development of insulin resistance [47] and liver tissue fibrosis [48]. In the present study, a significantly increased Elovl-6 and Elovl-5 index was present in T2DM rats, where liver steatosis was observed, but an increased Elovl-6 (but not Elovl-5) index was also present in the control group supplemented with saponins where fat content in hepatocytes was not noted, showing that estimated Elovl-6 or Elovl-5 indices are not suitable markers of steatosis.

There are reports indicating an increase in myristic acid (C14:0), palmitic acid (C:16:0), and oleic acid (C18:0) in NAFLD liver tissue in a mouse model [49], but this was not observed in the present study.

There is evidence suggesting that PUFAs omega-3 (n-3) help to improve glucose tolerance, insulin sensitivity and reduce the risk factors for metabolic syndrome [50]. The ingestion of PUFAs reduces the accumulation of liver lipids in animal models [51,52]. The synthesis of n-3 PUFA is small in adults, and that is why these fatty acids need to be present in the diet [53].

In our experiment, PUFA (18:3n-3 and 18:2n-6) and total PUFA were significantly decreased in the C-DM group compared to the nDM group. Supplementation with saponins or with the combination of saponins with inulin did not cause significant differences among non-diabetic groups. Despite the lack of significant differences after supplementation with saponins in the diabetic group, supplementation with the combination of saponins with inulin caused a decrease (on the border of significance) in 18:2n-6, 18:3n-3. An adverse experiment performed on fasted animals revealed that hepatic triacylglycerol was enriched with both n-3 and n-6 PUFA during fasting, so n-3 and n-6 PUFA did not come from dietary sources [54]. Short-term fasting caused a significant reduction in hepatic expression of Elovl6 and SCD1, but the hepatic expression of the key genes involved in the biosynthesis of n-3 and n-6 PUFA and expression of Fads1, Fads2, Elovl2, and Elovl5 were not changed in the fasted group suggesting that the enrichment of hepatic TAG PUFA was not an effect of increased biosynthesis [54]. However, a hepatic expression of fatty acid transporters that preferentially mediate uptake of n-3 and n-6 PUFA into cells such as Fabp7, Fatp2, Fabp1, and Acsl6 was increased in the fasted group compared with the fed group [54]. Marks et al. [54] concluded that selective uptake by the liver plays an important function in hepatic fatty acid composition that occurred with fasting.

An important parameter in the study of NAFLD is hepatic de novo lipogenesis (DNL), which is a part of complex metabolic pathways in the liver and depends on glycolysis and carbohydrate metabolism [55]. In hepatic insulin resistance, glucose metabolism in hepatocytes does not respond to insulin, but lipogenesis carries on, and triglyceride content increases. This is a kind of paradox in type 2 diabetes. This situation indicates that the state where the liver is resistant to insulin is associated with the regulation of DNL by insulin-dependent sterol regulatory element-binding protein 1c (SREBP-1c) [56]. There is evidence supporting the thesis that hepatic DNL is increased in humans with insulin resistance and in humans with NAFLD [57,58]. Such relations were partially observed in the present study, where the DNL index was significantly increased in rats with induced diabetes type 2. On the other hand, Vatner et al. [58] data support the thesis that NAFLD can develop independently on the action of insulin in the liver and also independently on the higher fat level in the liver [58].

There are many studies with different potential markers of NAFLD among liver fatty acids. A few of them are mentioned above, and it is evident that very often, data contradict each other, and the ideal marker of NAFLD is elusive. For example, elevated SFA and decreased levels of UFA (MUFA and PUFA), arachidonic acid, key n-3 fatty acid, and the (n-6)/(n-3) fatty acid ratio were observed in NAFLD [49,59]. Similar results were also present in the current study in the liver of C-DM rats, where liver steatosis was also noted. Surprisingly, similar results of the liver fatty acids (significantly elevated total SFA and decreased total of MUFA and the ratio PUFA/SFA) were also present in the non-diabetes group supplemented with saponins, but in this group, liver steatosis was not observed. Although liver fatty acids parameters were to some extent similar (decreased PUFA/SFA ratio, elevated Elovl-6 index) in these two groups and theoretically associated with liver steatosis, such morphology was present only in the C-DM group. The main difference was that the level of SCD18 index significantly decreased in the non-diabetes group supplemented with saponins and increased (though not significantly) in the C-DM group.

Stearoyl-CoA desaturase-1 (SCD-1) is one of the most important enzymes in fatty acid synthesis. It catalyzes the reaction of the introduction of the first *cis* double bond in the Δ9 position of saturated fatty acids (that are lipotoxic), stearic acid (C18:0) and palmitic acid (C16:0), to generate the less lipotoxic MUFAs; respectively, oleic acid (C18:1n-9) and palmitoleic acid (C16:1n-7) [60]. The overexpression of SCD is associated with metabolic disorders [61] and may lead to pathological changes in the liver as one of the adaptive mechanisms [62].

## 5. Conclusions

In summary, supplementation with 100 mg of TT saponins or saponins with inulin for one month decreased the level of lipid droplets deposited in the hepatocytes of rats with induced type 2 diabetes. Moreover, it also seemed to have favorable effects on the plasma lipid profile in the rats. However, in the non-diabetes group, additional supplementation with inulin had a negative effect on liver morphology (with a microvesicular type of steatosis). Moreover, supplementation with inulin had a negative effect on plasma glucose in both diabetic and non-diabetic rats. While the suspected effect of inulin should be positive, instead, it had a negative effect on the liver morphology of control rats. These data show that a diet enriched with fermentable fibers reveals different effects in various organisms, and not all sources and forms of fiber are beneficial to health and thus should be approached with great caution. Supplementation with TT saponins was associated with a significant decrease in the liver SCD-18 index in non-diabetes group. An additional conclusion is that SCD-18 index seems to have diagnostic potential as a marker of steatosis.

## 6. Limitations

There were six animals per subgroup according to the recommendations of the Local Ethical Committee on Animal Testing. This is a sufficient but minimal number of animals, so the study should be repeated with a larger number of animals in the subgroups.

## Figures and Tables

**Table 1:** Scores of hepatic steatosis and hepatic fibrosis staging.

Group	N (n)	Histological Grades of Steatosis Number of Evaluated Histological Fields (Percentage of Grade of Steatosis)				Fibrosis Stage (N)				
		0	1	2	3	0	1	2	3	4
c-C	6 (60)	60 (100)	0 (0)	0 (0)	0 (0)	6	0	0	0	0
C-Sap	6 (60)	60 (100)	0 (0)	0 (0)	0 (0)	6	0	0	0	0
C-Sap + IN	6 (60)	6 (10) ^b^	20 (33.3) ^c^	28 (46.7) ^c^	6 (10) ^b^	4	2	0	0	0
c-DM	6 (60)	0 (0)	3 (5)	44 (73.3)	13 (21.7)	1	4	1	0	0
DM-Sap	6 (60)	42 (70) *	13 (21.7) ^a^	5 (8.3) *	0 (0) *	5	1	0	0	0
DM-Sap + IN	6 (60)	53 (88.3) *	7 (11.7)	0 (0) *	0 (0) *	5	1	0	0	0

Steatosis data are expressed as counts and percentages (in parentheses). N: number of animals; (n): number of evaluated histological fields. * *p* < 0.0001 vs. c-DM, ^a^ *p* < 0.001 vs. c-DM, ^b^ *p* < 0.05 vs. c-C, ^c^ *p* < 0.0001 vs. c-C.

**Table 2:** The baseline plasma lipid profile, glycated hemoglobin, and glucose in each subgroup of rats.

Parameters	Control Groups			Diabetes Groups		
	c-C	C-Sap	C-Sap + IN	c-DM	DM-Sap	DM-Sap + IN
HbA1c/HbF (%)-IFCC	7.97 ± 0.55	8.15 ± 1.44	8.87 ± 1.46	8.38 ± 1.44	9 ± 1.92	10.08 ± 1.66
Glucose (mg/dL)	69.33 ± 4.93	69.33 ± 5.42	76 ± 5.48 ^b^	184.5 ± 46.48 *	252 ± 135.55	274.5 ± 64.78 ^∆^
Triglycerides (mg/dL)	39.83 ± 23.61	38.0 ± 13.18	48.33 ± 14.31	67.0 ± 35.39	40.0 ± 21.4	42.33 ± 17.99
Cholesterol (mg/dL)	59.0 ± 8.07	64.83 ± 8.08	61.83 ± 8.61	51.5 ± 9.07	46.83 ± 14.08	55.5 ± 19.89
HDL (mg/dL)	19.5 ± 1.97	18.5 ± 2.17	17.67 ± 2.25	16.83 ± 1.6 *	17.5 ± 7.26	18.67 ± 4.93
LDL (mg/dL)	9.0 ± 3.35	10.33 ± 2.73	9.0 ± 2.76	6.67 ± 2.94	7.5 ± 1.87	7.67 ± 3.44
Rate						
TC/HDL	2.04 ± 1.24	2.12 ± 0.87	2.78 ± 0.87	3.98 ± 2.02 ^b^	2.75 ± 1.81	2.59 ± 1.66

Data are expressed as mean ± SD of 6 rats in each group. * *p* ˂ 0.05 vs. c-C; ^b^ *p* < 0.055 vs. c-C on the border of statistically significant differences; ^∆^ *p* < 0.05 vs. c-DM.

**Table 3:** Liver fatty acid profiles in each subgroup of rats.

Fatty Acids (%)	Control Groups			Diabetes Groups		
	c-C	C-Sap	C-Sap + IN	c-DM	DM-Sap	DM-Sap + IN
C12:0 Lauric Acid	2.08 ± 0.34	2.03 ± 0.31	1.63 ± 0.12 *	2.58 ± 0.58	3.22 ± 1.17 ^b^	3.34 ± 0.39 ^Δ^
C14:0 Myristic	0.52 ± 0.25	0.50 ± 0.14	0.55 ± 0.19	0.52 ± 0.23	0.68 ± 0.03	0.75 ± 0.14 ^Δ^
C14:1 Myristoleic	0.36 ± 0.07	0.28 ± 0.03 *	0.33 ± 0.06	0.32 ± 0.02	0.35 ± 0.04	0.33 ± 0.07
C15:0 Pentadecanoic	0.21 ± 0.09	0.34 ± 0.12	0.46 ± 0.25	0.12 ± 0.02	0.4 ± 0.96	0.12 ± 0.02
C16:0 Palmitic	22.82 ± 0.73	23.24 ± 0.79	23.51 ± 0.81	21.29 ± 1.33 *	20.71 ± 5.94	21.68 ± 0.53
C16:1n7 Palmitoleic	0.41 ± 0.1	0.36 ± 0.08	0.53 ± 0.12	0.28 ± 0.13	0.29 ± 0.12	0.24 ± 0.08
C17:0 Heptadecanoic	0.67 ± 0.08	0.66 ± 0.08	0.55 ± 0.07 *	0.36 ± 0.02 *	0.44 ± 0.03 ^Δ^	0.46 ± 0.05 ^Δ^
C18:0 Stearic	18.14 ± 2.2	21.08 ± 0.5 *	17.98 ± 4.17	22.13 ± 44.44	22.98 ± 10.33	22.49 ± 1.09
C18:1n9 Oleic	11.57 ± 2.31	9.03 ± 0.54 *	10.96 ± 2.42	15.88 ± 38.57	14.16 ± 20.81	13.92 ± 2.24
C18:1n-7 Vaccenic	4.1 ± 0.21	3.38 ± 0.28 *	3.55 ± 0.13 *	2.47 ± 0.88	2.27 ± 0.46	2.28 ± 0.32
18:2n-6 Linoleic	18.81 ± 0.92	17.77 ± 0.94	20.22 ± 2.58	13.66 ± 1.1 *	12.51 ± 5.42	12.31 ± 0.95 ^b^
18:3n-6 γ-Linoleic	0.19 ± 0.05	0.23 ± 0.07	0.18 ± 0.07	0.13 ± 0.03	0.14 ± 0.01	0.13 ± 0.04
18:3n-3 Linolenic	0.77 ± 0.13	0.63 ± 0.1	0.87 ± 0.21	0.27 ± 0.1 *	0.19 ± 0.04	0.16 ± 0.02 ^b^
C20:0 Arachidic	0.31 ± 0.07	0.26 ± 0.03	0.24 ± 0.03	0.2 ± 0.01	0.21 ± 0.01	0.18 ± 0.03
C20:1 11-Eicosenoic	0.33 ± 0.16	0.23 ± 0.13	0.4 ± 0.31	0.08 ± 0.01	0.1 ± 0.01	0.09 ± 0.03
C20:2n6 Eicosadienoic	0.57 ± 0.11	0.57 ± 0.1	0.4 ± 0.11*	0.33 ± 0.06	0.28 ± 0.02	0.28 ± 0.09
C20:3n6 Eicosatrienoic	0.59 ± 0.07	0.61 ± 0.12	0.51 ± 0.18	0.75 ± 0.1	0.73 ± 0.25	0.78 ± 0.13
C20:4n6 Arachidonic	10.83 ± 1.33	12.69 ± 1.88	11.03 ± 1.59	11.64 ± 39.83	14.33 ± 14.45	13.8 ± 1.52
C20:5n3 Eicosapentaenoic	0.43 ± 0.12	0.34 ± 0.06	0.49 ± 0.12	0.22 ± 0.03	0.22 ± 0.03	0.19 ± 0.04
C22:4n6 Docosatetraenoic	0.63 ± 0.18	0.47 ± 0.11	0.52 ± 0.12	0.55 ± 0.04	0.47 ± 0.12	0.43 ± 0.06
C22:5n3 Docosapentaenoic	1.21 ± 0.47	1,0 ± 0.36	1.13 ± 0.35	1.04 ± 1.3	0.63 ± 0.1	0.77 ± 0.16
C22:6n3 Docosahexaenoic	4.44 ± 0.69	4.31 ± 0.81	3.98 ± 0.61	5.17 ± 5.69	4.49 ± 2.86	5.3 ± 1.08
**Parameter**						
∑-SFA	44.74 ± 3.05	48.12 ± 0.56 *	44.92 ± 4.15	47.2 ± 2.45	48.64 ± 1.44	49.01 ± 0.93
∑-PUFA	38.4 ± 0.75	38.61 ± 0.66	39.31 ± 1.65	33.76 ± 2.26 *	34.19 ± 2.06	34.13 ± 1.9
∑-MUFA	16.78 ± 2.83	13.27 ± 0.54 *	15.77 ± 2.66	19.04 ± 3.25 *	17.16 ± 2.48	16.86 ± 2.78
PUFA/SFA	0.86 ± 0.07	0.8 ± 0.02 ^c^	0.88 ± 0.12	0.72 ± 0.06 *	0.7 ± 0.05	0.7 ± 0.03
∑-n-6	31.62 ± 0.87	32.34 ± 1.68	32.85 ± 1.42	27.06 ± 1.77 *	28.47 ± 1.74	27.71 ± 1.35
∑-n-3	6.86 ± 0.97	6.27 ± 1.2	6.46 ± 0.56	6.7 ± 0.83	5.72 ± 0.86	6.42 ± 1.15
n-6/n-3	4.71 ± 0.47	5.36 ± 1.27	5.11 ± 0.44	4.08 ± 0.47	5.06 ± 0.76 ^Δ^	4.44 ± 0.89
DNL index	1.22 ± 0.08	1.31 ± 0.08	1.17 ± 0.12	1.56 ± 0.07 *	1.66 ± 0.14	1.77 ± 0.16
SCD16 index	0.02 ± 0.005	0.02 ± 0.004	0.02 ± 0.005	0.01 ± 0.007	0.01 ± 0.007	0.01 ± 0.004
SCD18 index	0.66 ± 0.23	0.43 ± 0.04 *	0.68 ± 0.37	0.75 ± 0.26	0.62 ± 0.13	0.62 ± 0.14
∆6D index	0.01 ± 0.003	0.01 ± 0.004	0.009 ± 0.003	0.01 ± 0.002	0.01 ± 0.004	0.01 ± 0.004
Elovl-5 index	3.38 ± 0.89	2.96 ± 1.14	3.35 ± 2.07	5.71 ± 1.73 *	5.03 ± 0.95	7.44 ± 4.92
Elovl-6 index	0.79 ± 0.09	0.91 ± 0.05 *	0.77 ± 0.22	1.05 ± 0.18 *	1.11 ± 0.12	1.04 ± 0.08
Elongation FA index	1.59 ± 0.48	1.3 ± 0.05	1.24 ± 0.14	1.79 ± 0.1	1.8 ± 0.1	1.68 ±0.06 ^Δ^

Data are expressed as mean ± SD of 6 rats in each group. * *p* < 0.05 vs. c-C, ^Δ^ *p* < 0.05 vs. c-DM, ^b^ *p* < 0.055 vs. c-DM and ^c^ *p* < 0.055 vs. c-C on the border of statistically significant differences.

## Data Availability

The data presented in this study are available on request from the corresponding author.
